# Influence of Luteolin on Physicochemical Characteristics, Structural Changes and Functional Properties of Casein Fermentation System

**DOI:** 10.3390/foods15112015

**Published:** 2026-06-04

**Authors:** Wanying Zhang, Haibo Lu, Yueyuan Lu, Yang Sun, Guojun Du, Yue Zhao, Yonghui Sun, Nazi Yang, Liying Bo, Jian Ren, Jingjing An, Meng Wang

**Affiliations:** 1 Faculty of Food Quality and Safety, Qiqihar University, Qiqihar 161006, China; 18745150721@163.com (W.Z.); renjian1970789@163.com (J.R.); 2 Tianjin Institute of Industrial Biotechnology, Chinese Academy of Sciences, Tianjin 300308, Chinasunyh@tib.cas.cn (Y.S.); yangnz26@tib.cas.cn (N.Y.);; 3 Haihe Laboratory of Synthetic Biology, Tianjin 300308, China

**Keywords:** luteolin, casein, in vitro protein digestibility, antioxidant capacity, functional nutritional factors

## Abstract

As a core nutritional component of milk, casein features excellent digestibility and biocompatibility, making it an ideal carrier for embedding natural bioactive substances in dairy product research. Luteolin, a typical flavonoid compound with superior antioxidant and anti-inflammatory bioactivities, is limited in industrial dairy applications due to poor environmental stability and low biological utilization. Moreover, the dynamic interplay mechanism between luteolin and casein throughout fermentation and cold storage remains unclear. This study hypothesized that luteolin could assemble with casein via non-covalent binding to form stable composite fermentation system, thereby optimizing the overall quality and functional attributes of fermented milk. This work aimed to explore the binding characteristics of luteolin of casein in fermented milk and its regulatory effects on products’ physicochemical properties, antioxidant capacity and nutritional digestibility. Experimental outcomes verified the hypothesis that luteolin bonded with casein through hydrogen bonding and hydrophobic interactions. With increased luteolin supplementation, the fermentation system presented lowered pH and elevated titratable acidity. Compared with control fermentation system without luteolin, the fermentatiuon system containing 0.06% luteolin achieved 31.31% higher DPPH radical scavenging rate, 27.02% higher ABTS clearance capacity, and 26.42% higher in vitro protein digestibility (*p* < 0.05). Dose-dependent increases in particle size and absolute zeta-potential enhanced system colloidal stability, while FTIR detection confirmed obvious variations in protein secondary structure in fermented milk. This study elucidates the distinctive structure–function correlation of the luteolin–casein fermentation system in fermented dairy matrices, providing original insights and reliable theoretical support for developing novel dairy products rich in functional nutritional factors.

## 1. Introduction

Milk protein represents a vital source of high-quality protein in the human diet and is widely employed in food manufacturing. Bovine milk generally contains approximately 3.3% (*w*/*w*) protein, comprising hundreds of distinct individual proteins. These milk proteins are mainly classified into two fractions: casein, accounting for nearly 80% of total protein, and whey protein, making up the remaining 20% [1,2]. As the predominant protein fraction, casein has attracted extensive research interest in food processing owing to its superior nutritional value and versatile functional attributes. Beyond providing all essential amino acids for human growth and maintenance, milk protein exhibits a range of bioactivities, such as immunomodulatory, antioxidant, and lowering effects [2,3]. Furthermore, milk proteins possess valuable techno-functional properties, water-holding capacity, and oil-binding capacity, which render them indispensable ingredients in food formulation and product development.

In the food industry, milk proteins are widely utilized in the formulation of dairy products, sports nutritional foods, and a variety of functional food matrices. The non-covalent assembly of protein–polyphenol complexes has been proven effective in enhancing the emulsifying properties, gelling behavior, and oxidative stability of food systems [4,5]. Nevertheless, native food proteins usually possess relatively low bioactivities, especially antioxidant activity, prior to enzymatic hydrolysis or microbial fermentation into bioactive peptides [6]. Accordingly, the development of composite formulations via the interfacial associations between proteins and polyphenols has emerged as a cutting-edge strategy to enhance the functional attributes and nutritional value of food products. Polyphenols, distinguished by their complex structural diversity and potent bioactivities, are regarded as essential health-promoting components in modern functional food design. Consequently, they have attracted considerable scientific attention due to their robust antioxidant, anti-inflammatory, and antimicrobial properties, as well as their potential to alleviate the progression of chronic metabolic disorders [7,8,9]. Despite their multifaceted bioactivities, the practical implementation of polyphenols within industrial food systems remains significantly hampered by inherent physicochemical constraints. Specifically, their inadequate aqueous solubility, marginal stability under variable environmental conditions, and restricted bioavailability render them highly susceptible to degradation during conventional processing practices, including thermal treatment or pH adjustment, thereby undermining their functional efficacy in real-world food matrices [10,11,12,13]. To surmount these limitations, investigators have explored the conjugation of polyphenols with biological macromolecules (e.g., milk proteins) to form colloidal complexes or encapsulation matrices, with the primary objective of enhancing their solubility, physicochemical stability, and bioaccessibility [14,15,16,17]. For instance, Ferraro et al. [18] demonstrated that rosemary acid interacts with casein micelles through reversible physical associations, resulting in the formation of a structurally stable complex in dispersion, particularly at an acidic pH of 3. Furthermore, Chen et al. [19] reported a significant enhancement in the free-radical scavenging activity of a coffee–milk system, which increased from 33.55% to 49.37% upon the addition of 90 mg/mL of milk protein. In a separate study, Li et al. [20] observed that the incorporation of polyphenols into lactoferrin significantly ameliorated its foaming capacity and stability (*p* < 0.05), as evidenced by the markedly improved foam expansion rate and persistence compared to the untreated control.

Luteolin, a ubiquitous natural flavonoid, is abundantly distributed in diverse plant sources including celery, broccoli, peppers, oranges, and various medicinal herbs [21]. For centuries, luteolin-rich plants have been used in traditional Chinese medicine to alleviate hypertension, inflammatory disorders, and cancer-related symptoms. Modern studies have extensively documented its multifunctional bioactivities, such as anti-inflammatory, antioxidant, anticancer, neuroprotective, cardioprotective, and metabolic regulatory effects [22,23,24]. Nevertheless, the practical application of luteolin in food systems is severely hindered by its intrinsic drawbacks. Luteolin exhibits poor aqueous solubility, insufficient stability against heat, light, and pH fluctuations, and low oral bioavailability, which lead to rapid degradation during food processing and limited physiological efficacy [25,26]. Moreover, most current studies remain at the in vitro or animal model stage, with insufficient data on structure–activity relationships and dose-dependent effects in real food matrices, creating barriers to its industrial translation [27,28].

Therefore, systematic exploration of the formation mechanism, structural features, and functional performance of casein–luteolin complexes will not only deepen the understanding of protein–polyphenol interaction rules but also lay a theoretical foundation for developing efficient and stable functional composite ingredients, which is of great significance for promoting innovative applications in health foods. This study investigates the effects of adding 0.02%, 0.04%, 0.06%, and 0.09% of luteolin on the physicochemical properties, rheological behavior, texture, antioxidant capacity, and in vitro digestibility of casein–luteolin fermentation system in a control group without adding luteolin, with the aim of determining the optimal luteolin loading level.

## 2. Materials and Methods

### 2.1. Materials and Reagents

Commercial casein powder was kindly supplied by Jiangsu Jianyun Biotechnology Co., Ltd. (Jiangyin, China). Whey protein isolate was obtained from Hilmar Cheese Inc. (Hilmar, CA, USA); ingredients include protein, fat, carbohydrates, sodium, and vitamin B6, while lactose was purchased from Leprino Foods Inc. (Denver, CO, USA). Luteolin (Purity ≥ 98%) was procured from Shanghai McLyn Biochemical Technology Co., Ltd. (Shanghai, China). The freeze-dried starter culture, comprising *Streptococcus thermophilus* (CGMCC-1.1878) and *Lactobacillus delbrueckii* subsp. *bulgaricus* (CICC-6047), was purchased from Beijing Chuanxiu Technology Co., Ltd. (Beijing, China). All other chemicals and reagents utilized in this study were of analytical grade and used without further purification.

### 2.2. Preparation of Luteolin–Casein Complex System

These ingredients contribute to forming the fundamental gel structure of yogurt, constructing the gel skeleton, elevating total protein content, and supplying carbon sources for lactic acid bacteria fermentation. Initially, the mixed pre-fermentation system was prepared by dissolving 3.2% (*w*/*w*) casein powder, 2.5% (*w*/*w*) whey protein isolate and 2.6% (*w*/*w*) lactose in ultrapure water at 42 °C [29]. Afterwards, luteolin was supplemented to the system to reach final concentrations of 0.02%, 0.04%, 0.06% and 0.09% (*w*/*v*), designated as the experimental groups. The blank control group contained no luteolin.

All prepared samples were subjected to high-pressure homogenization using a Panda Plus 2000 homogenizer (GEA Niro Soavi S.p.A., Parma, Italy) at a pressure of 100 MPa, followed by thermal processing via pasteurization at 90 °C for 10 min. Following heat treatment, the samples were immediately cooled to 42 °C and sealed with sterile film to maintain aseptic conditions.

Fermentation was initiated by the inoculation of 0.125% (*w*/*v*) starter culture, comprising *Streptococcus thermophilus* and *Lactobacillus delbrueckii* subsp. *bulgaricus*. A control sample without luteolin was prepared alongside the treatment groups and subjected to an identical processing workflow to eliminate confounding variables derived from procedural differences. After fermentation, all samples were immediately transferred to the refrigerator and stored at 4 °C for 24 h before further analysis.

### 2.3. Preparation of All Fermented Samples

The preparation of fermented milk samples rich in luteolin and the control fermented milk samples without luteolin were fabricated using a procedure adapted with minor revisions from the protocol reported by Megrius et al. [30]. The inoculated dispersions were portioned into 50 mL beakers, securely sealed, and subjected to isothermal fermentation at 42 °C for 8 h. Upon completion of fermentation, all samples were promptly transferred to 4 °C storage and held for 24 h to achieve structural equilibration prior to further experimental measurements.

All samples were subjected to storage treatment and periodic performance analysis in this study. After preparation, the samples were placed in a medical-grade electronic temperature-controlled refrigerator (Model: BCD-400EGX5S, Anhui Konka Tongchuang Electric Appliance Co., Ltd., Chuzhou, China) and stored at 4 ± 1 °C under dark conditions throughout the whole experimental period. The samples were packaged in 50 mL glass beakers with a wall thickness of 0.5 mm, which were pre-sterilized by ultraviolet irradiation for 3 h before sample loading. Each beaker was filled with 40 mL of sample and tightly sealed with plastic wrap to prevent moisture evaporation and external microbial contamination. Sample collection was carried out on days 1, 7, 14, and 21 of storage for the determination of various quality indicators. To minimize sampling bias and guarantee experimental reproducibility, three independent replicates were set for each time point, and all collected samples were analyzed immediately. All storage and sampling operations were performed under strict aseptic conditions.

### 2.4. Detection of pH and Titratable Acidity of All Samples

Samples were collected on days 1, 7, 14, and 21 of refrigerated storage for pH and titratable acidity determination. The pH value of each sample was measured at ambient temperature using a calibrated digital pH meter (Sartorius Scientific Instruments, Beijing, China) via direct immersion of the electrode.

Titratable acidity was quantified using a modified method established by Parvarei et al. [31]. In brief, 5 g of sample was homogenized with 20 mL of deionized water, followed by dropwise addition of 0.5% (*w*/*v*) phenolphthalein indicator. The mixture was then titrated with 0.1 N NaOH solution until a faint pink color persisted for 30 s. The volume of NaOH consumed was recorded and expressed as milliliters per 100 g of sample (mL/100 g).

### 2.5. Colorimetric Analysis

All samples were subjected to color evaluation employing the CIE Lab color system* as previously described [32]. Colorimetric parameters including lightness (L*), redness-greenness (a*), and yellowness-blueness (b*) were determined using a colorimeter (CR-400; Konica Minolta Inc., Tokyo, Japan) fitted with a CR-A70 liquid sample attachment. Measurements were performed under a D65 standard light source and 10° standard observer angle at a controlled temperature of 12 ± 2 °C.

### 2.6. Evaluation of Syneresis in All Samples

Syneresis of the samples was quantified following the method described by Varnaitė et al. [33] with minor modifications. A 5 mL aliquot of each sample was transferred into a 10 mL centrifuge tube and centrifuged at 2000× *g* for 20 min at room temperature using an MPW 260R centrifuge (MPW Med. Instruments, Warszawa, Poland). The supernatant was carefully collected and weighed. Calculate the syneresis rate using Equation (1):

(1) $$\mathrm{Syneresis}\;(\%)=\frac{\mathrm{Weight}\;\mathrm{of}\;\mathrm{supernatant}}{Weight\;\mathrm{of}\;\mathrm{sample}}\times 100$$

### 2.7. Texture Evaluation of All Samples

Texture profiling was performed using a modified approach adapted from Cheng et al. [34]. Measurements were conducted at ambient temperature with a QTS-25 texture analyzer (AMETEK Brookfield, Middleboro, MA, USA) equipped with a 35 mm cylindrical probe. A double-puncture test mode was employed under the following conditions: test speed 1 mm/s, trigger force 0.15 N, and penetration depth 30 mm (corresponding to 50% sample compression).

### 2.8. Rheological Determination of All Samples

Rheological properties were analyzed using a Kinexus Pro^+^ rotational rheometer (Malvern Instruments, Worcestershire, UK) as referenced [35]. The instrument was equipped with a titanium-alloy parallel-plate geometry featuring a 40 mm diameter, 4° cone angle, and 0.15 mm measuring gap. Dynamic rheological properties were determined via frequency sweeps from 0.1 to 10 Hz at a constant strain of 0.5%, which lies within the linear viscoelastic range. Storage modulus (G′) and loss modulus (G″) at 1 Hz were automatically recorded. Apparent viscosity was further measured under steady shear conditions across a range of 0.1 to 100 s^−1^.

### 2.9. Assessment of Radical Scavenging Activity

DPPH radical scavenging activity was determined using a modified method from Trigueros et al. [35]. Briefly, 2.0 mL of 0.1 mmol/L DPPH ethanolic solution was mixed thoroughly with 2.0 mL of diluted sample solution (2 mg/mL), followed by incubation in the dark for 30 min. The absorbance at 517 nm was measured and recorded as A_i_. For the blank control, 2.0 mL of anhydrous ethanol was mixed with 2.0 mL of sample solution (2 mg/mL), incubated under identical dark conditions for 30 min, and the absorbance was recorded as A_z_. For the negative control, 2.0 mL of anhydrous ethanol was mixed with 2.0 mL of DPPH solution, incubated in the dark for 30 min, and the absorbance was recorded as A_j_. All absorbance readings were acquired at 517 nm. The DPPH radical scavenging rate is calculated according to (2):

(2) $$\mathrm{DPPH}\;\mathrm{Clearance}\;\mathrm{Rate}\;\%=[1-\frac{A_{i}{-A}_{z}}{A_{j}}]\times 100\%$$

ABTS radical cation scavenging activity was determined following a method adapted from Cheng et al. [34]. Briefly, 2.0 mL of ABTS working solution was mixed with 2.0 mL of anhydrous ethanol, reacted in darkness for 10 min, and the absorbance at 734 nm was measured and recorded as A_0_. Next, 2.0 mL of ABTS working solution was combined with 2.0 mL of sample solution, mixed thoroughly, and incubated in the dark for 10 min. The absorbance at 734 nm was then recorded as A. The ABTS radical scavenging rate is calculated according to (3):

(3) $$\mathrm{ABTS}\;C\mathrm{learance}\;\mathrm{Rate}\;\%=[1-\frac{A_{0}-A}{A_{0}}]\times 100\%$$

### 2.10. In Vitro Protein Digestibility Assessment

The in vitro protein digestibility of casein–luteolin complexes was determined using a modified method from Ren et al. [36]. Briefly, sample pH was adjusted to 2.0, followed by addition of 10 mL simulated gastric fluid. The mixture pH was re-adjusted to 2.0 using 0.5 M NaOH, then incubated in a thermostatic water bath at 37 °C with shaking at 110 rpm for 2 h. For the control group, simulated gastric fluid was replaced with an equal volume of deionized water. Digestion was terminated by adding 1 mL of 10% (*w*/*v*) trichloroacetic acid (TCA) and mixing thoroughly. The digestate was centrifuged at 4000× *g* for 15 min at 20 °C, and the supernatant was collected and diluted to 100 mL. Protein content was quantified by the Bradford method [37]. A total of 0.5 mL of supernatant was transferred to a 10 mL colorimetric tube, mixed with 5 mL Coomassie Brilliant Blue G-250 reagent, and held for 2 min. Absorbance was measured at 595 nm. Calculate protein digestibility using Equation (4):

(4) $$\mathrm{Digestibility}\;\left(\%\right)=\frac{\mathrm{Soluble}\;\mathrm{protein}\;\mathrm{in}\;\mathrm{digesta}}{\mathrm{Total}\;\mathrm{protein}\;\mathrm{in}\;\mathrm{initial}\;\mathrm{sample}}$$

### 2.11. Particle Size and Zeta Potential of Samples

Particle size distribution and zeta potential were determined following a modified method reported by Li et al. [38]. Samples were diluted to 1% (*w*/*v*) with deionized water and adjusted to pH 7.0 prior to measurement. Tests were performed at 25 °C using a Mastersizer 2000 laser diffraction analyzer (Malvern Instruments Ltd., Malvern, UK), with a protein refractive index of 1.46 and water as the dispersant.

### 2.12. FT-IR Spectrum Analysis

FTIR spectra were acquired using a modified procedure from Li et al. [39]. Freeze-dried composites were ground into fine powders, then analyzed using a Fourier transform infrared spectrometer (Thermo Fisher Scientific Inc., Waltham, MA, USA) equipped with an attenuated total reflection (ATR) accessory. Spectra were recorded over the wavenumber range of 4000–400 cm^−1^ at a resolution of 4 cm^−1^, averaging a minimum of 64 scans.

### 2.13. Statistical Analysis

Statistical analysis was performed using SPSS software (Version 26.0; SPSS Inc., Chicago, IL, USA). All experiments were carried out in triplicate independently, and data were presented as mean ± standard deviation (SD). Intergroup differences were analyzed by two-way analysis of variance (ANOVA, MATLAB, R2024b) followed by LSD post hoc multiple comparisons. Differences were considered statistically significant at *p* < 0.05.

## 3. Results and Discussion

### 3.1. Changes in pH and Titratable Acidity (TA) of All Samples

According to Table 1, the concentration and storage time of luteolin exert a remarkable influence on the pH value and TA of the sample (*p* < 0.05). In detail, as these two factors increase, the acidity of the fermented sample rises gradually, which is manifested by a continuous drop in pH value and a corresponding gain in TA value.

On day 1 of storage, the pH value declined gradually with the elevation of luteolin concentration. This phenomenon was mainly attributed to the microenvironmental changes induced by the binding between luteolin phenolic hydroxyl groups and protein hydrophobic domains. The translocation of luteolin from protein hydrophobic regions to hydrophilic surfaces promoted the dissociation of partial phenolic hydroxyl groups, thereby releasing additional protons and reducing system pH [40]. During fermentation, lactic acid bacteria and other microorganisms in the system utilize carbon sources like lactose for metabolism, generating organic acids such as lactic acid and acetic acid. The buildup of these acidic substances directly results in the decline of pH value and the elevation of acidity [36]. Instead, molecular interactions in this system are dominated by reversible non-covalent forces, primarily hydrogen bonding and hydrophobic interactions. The abundant phenolic hydroxyl groups on polyphenol molecules act as hydrogen bond donors, effectively associating with carbonyl groups (C=O) in protein peptide bonds and polar side-chain residues. Since phenolic hydroxyl groups are rarely oxidized or depleted under acidic conditions, numerous active sites remain available to promote protein–polyphenol hydrogen bonding, which ultimately elevates system acidity [40]. At the same time, this interaction may also alter the protein charge characteristics as well. This further exerts a major effect on the acidification rate and key acidity of the polyphenol–casein complex fermentation system, directly affecting the fermentation process [41].

### 3.2. Color Changes in All Samples

Color is a key indicator affecting consumers’ sensory acceptance of food, directly related to the market attractiveness of the product. Table 2 summarizes the effects of adding different concentrations of luteolin on the color parameters (L*, a*, b*) of the luteolin–casein complex fermentation system during refrigeration. Compared with the control sample, the L* value (brightness) and a* value (redness) of samples with added luteolin were significantly reduced during storage (7–21 d) (*p* < 0.05), while the b* value (yellowness) was significantly increased (*p* < 0.05), and showed a concentration dependence. The decrease in the L* value may be attributed to the complex formed through the covalent interaction between luteolin and casein. The decrease in a* value is mainly affected by two factors. On the one hand, luteolin itself barely contributes to red color development. On the other hand, luteolin undergoes structural changes in the microstructural system of casein. In addition, natural red pigments such as anthocyanins in most fermented dairy products are gradually degraded during storage, which further results in the reduction in a* values [42]. The increase in b* value is primarily attributed to the inherent pale yellow characteristics of luteolin as a flavonoid compound. With the extension of refrigerated storage time, both a* and b* values of all samples decreased at the late storage stage (day 21), which could be explained by the degradation of luteolin under acidic conditions and long-term storage environments. This structure–color coupling change suggests that the color stability of the luteolin–casein complex is not only a problem of chemical degradation, but also closely related to the physical integrity of gel matrix. Therefore, when developing functional fermented dairy products rich in flavonoids, it is necessary to balance the molecular cross-linking strength and the structural maintenance ability under long-term storage environment to prevent negative impact of color deterioration on the sensory quality of the product.

### 3.3. Analysis on Syneresis Changes in All Fermenrted Samples

Syneresi behavior is a key indicator that reflects the water-holding capacity of protein gel networks, and its variation is closely related to the compactness of the three-dimensional gel structure [43]. As shown in Table 3, the syneresis rate of the composite system decreased significantly with the increase in luteolin level (*p* < 0.05). Compared with the control group, the samples supplemented with 0.06% and 0.09% luteolin presented a remarkable reduction in syneresis (*p* < 0.05). Luteolin binds to casein and functions as a molecular bridge, while hydrogen bonds further stabilize the interaction. Consequently, the internal moisture is better immobilized, and the water-holding performance of the composite system is effectively improved [44]. With the extension of storage time, the syneresis rate gradually increased, indicating a shift in the dynamic balance of polyphenol–protein binding during long-term storage. Some non-covalent interactions may dissociate or rearrange, leading to the relaxation of the network structure. Luteolin may undergo slow oxidative degradation in the acidic fermentation environment, which weakens its stabilizing effect on the protein network [45]. Further analysis showed that the inhibition of luteolin-induced dehydration shrinkage was not only due to the increase in gel density, but also related to the change in network topology. Through multi-point hydrogen bonding and possible non-covalent cross-linking, luteolin transforms the casein network from “coarse chains” to “fine cross-linking”, significantly reducing pore size distribution and enhancing capillary binding force, thereby more effectively fixing water.

### 3.4. Analysis of Textural and Rheological Properties of All Fermented Samples

Texture properties are pivotal factors governing the quality and consumer acceptability of fermented protein-based foods. Table 4 illustrates the concentration-dependent influence of luteolin on the texture parameters of the fermented samples during refrigerated storage. In comparison with the control fermented sample, the supplementation of 0.02%, 0.04%, and 0.06% luteolin significantly enhanced the hardness, springiness, and adhesiveness of the samples throughout the storage period (*p* < 0.05). With the elevation of luteolin concentration, these polyphenol molecules can more efficiently occupy the free volume within the complex or form extra cross-linking nodes, thereby reinforcing the rigidity of the complex’s network structure [46]. Nevertheless, when the luteolin concentration increased to 0.09%, a decrease in hardness was observed (though the value remained higher than that of the control group). This phenomenon is attributable to the “two-phase effect” inherent in polyphenol–protein interactions. Luteolin can facilitate protein cross-linking and fortify the gel network via multi-point binding; however, an excessively high concentration may result in the saturation of binding sites on the protein surface, or even induce local over-aggregation or co-precipitation. Such occurrences compromise the uniformity of the gel network and diminish its strength [47]. The reduction in hardness, springiness, and adhesiveness of the complex in the late storage stage is explained by the fact that long-term storage diminishes the activity of lactic acid bacteria, which inhibits further acidification. Meanwhile, the accumulation of lactic acid disrupts the interactions among protein molecules. The texture control of luteolin is also related to the plastic deformation ability of the gel network in the process of stress. The dense cross-linked network formed by moderate (≤0.06%) luteolin can evenly distribute stress and exhibit higher elastic recovery and adhesion work during compression; at a concentration of 0.09%, the area with excessive local aggregation will become a stress concentration point, and micro rupture will occur preferentially under stress, manifested macroscopically as a decrease in hardness. The continuous decline in hardness and elasticity during storage is not only related to the accumulation of lactic acid and non-covalent bond dissociation, but also to the competitive binding of degradation products of magnolol. These products cannot effectively bridge casein molecules, gradually weakening the energy dissipation ability of the network and ultimately reducing the overall stability of the texture [47].

**Figure 1:**
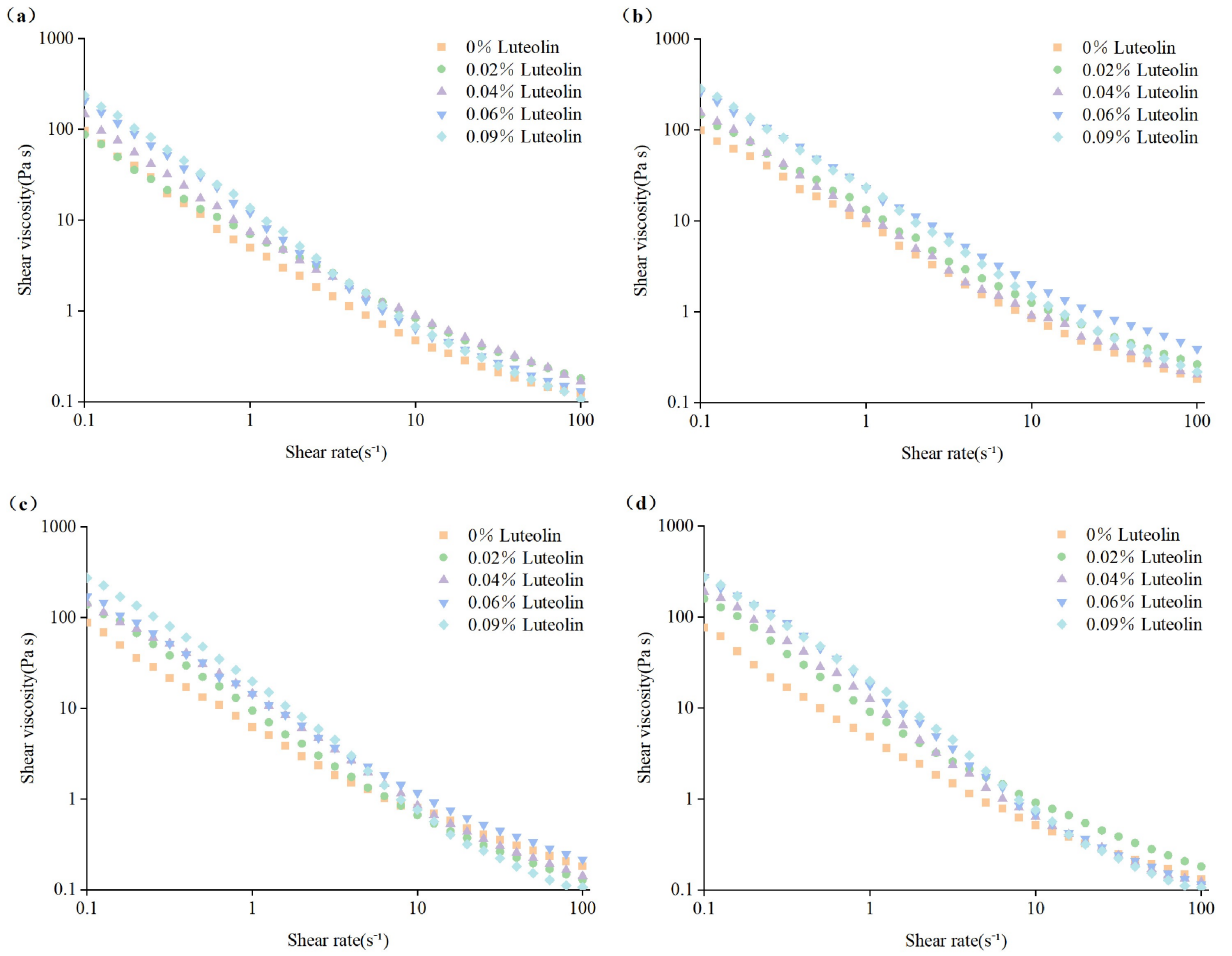
Apparent viscosity changes in all fermented samples assayed at 1 (**a**), 7 (**b**), 14 (**c**) and 21 days (**d**).

**Figure 2:**
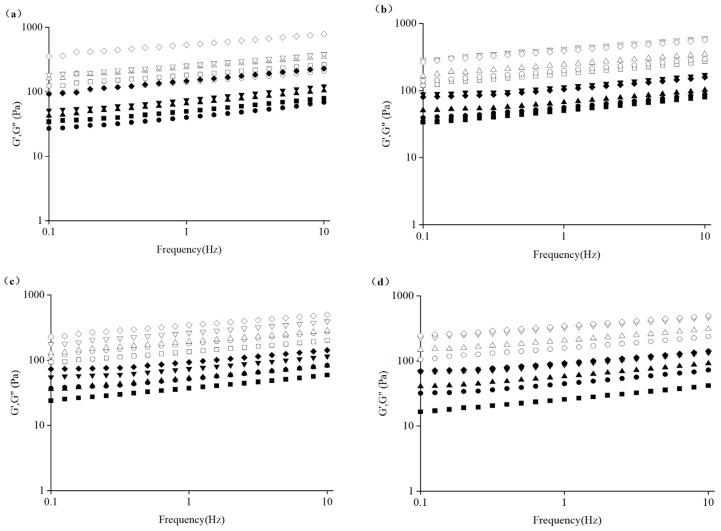
Storage modulus (G′) and loss modulus (G″) of all fermented samples measured at 1 (**a**), 7 (**b**), 14 (**c**), and 21 days (**d**). G′: Control sample without luteolin (○), the sample with 0.02% Luteolin (□), the sample with 0.04% Luteolin (△), the sample with 0.06% Luteolin (▽) and the sample with 0.09% Luteolin (◇). G″: Control sample without luteolin (●), the sample with 0.02% Luteolin (■), the sample with 0.04% Luteolin (▲), the sample with 0.06% Luteolin (▼), and the sample with 0.09% Luteolin (◆).

Given the critical role of rheological properties in enhancing food quality and optimizing nutritional characteristics, the influence of different luteolin concentration on the rheological behaviors (such as G′, G″, and apparent viscosity) of the fermented samples was evaluated. The apparent viscosity of all samples decreased with increasing shear rate, indicative of typical shear-thinning behaviour. As listed in Figure 1, samples containing 0.09% luteolin possessed higher apparent viscosity relative to the remaining formulations. As also illustrated in Figure 2, within the frequency scanning range of 0.1–10 Hz, the storage modulus (G′) of all samples was consistently higher than the loss modulus (G″), which is a typical characteristic of a gel network structure. This finding demonstrates that the composites formed a continuous and stable polymer network, exhibiting the rheological behavior of viscoelastic solids. Furthermore, this result confirms that the casein–luteolin complex underwent a transition from a viscoelastic liquid to a stable viscoelastic gel during the fermentation process. These outcomes indicate that the hydrophobic groups (e.g., aromatic rings) in the molecular structure of luteolin can bind to the hydrophobic regions of proteins through hydrophobic interactions. This binding helps reduce the exposure of internal hydrophobic groups of proteins to the aqueous phase, thereby forming a more ordered and compact three-dimensional network, which significantly enhances the rheological stability of the system [48,49].

### 3.5. Antioxidant Activity Evaluation of All Fermented Samples

**Figure 3:**
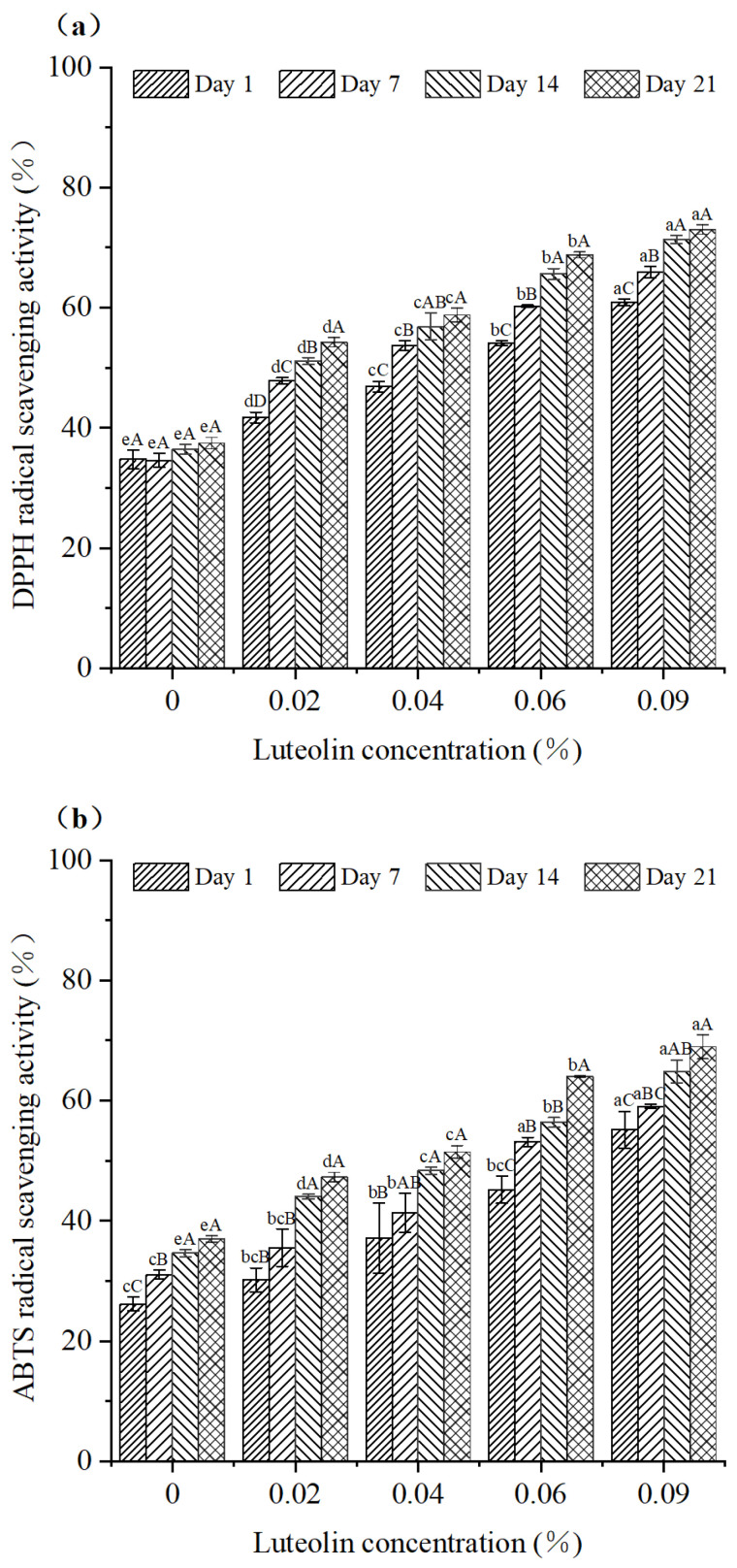
Changes in fermented sample antioxidant activity during storage. (**a**) DPPH radical scavenging activity; (**b**) ABTS radical scavenging activity. Note: Data were analyzed by two-way ANOVA. Values with different letters in the figure are significantly different (*p* < 0.05). The control sample is without luteolin.

Figure 3a depicts the DPPH radical scavenging activity of all fermented samples. In comparison to the fermented sample without luteolin supplementation, the samples with added luteolin (≥0.02%) exhibited higher scavenging activity, which continued to increase over the storage period. On the first day, the free radical scavenging activity of the 0.06% luteolin group (54.07%) was higher than that of the control group (34.76%). The scavenging activity of all groups continued to rise, achieving the maximum improvement relative to the control group (37.48%) on day 21 (0.06% group, 68.79%). This finding indicates that the addition of higher concentrations of polyphenols is conducive to enhancing the antioxidant capacity. Recent research further suggests that the higher the hydroxylation level of polyphenols, the stronger their binding affinity with proteins, thereby forming a more stable complex system and displaying superior DPPH radical scavenging ability [50,51]. From the perspective of synergistic effects, the combination of luteolin and casein does not result in functional superposition, but rather forms a synergistic enhancement system of “protection anchoring sustained release”. The three-dimensional network structure of casein provides a physical barrier for luteolin, which is stably anchored in the gel matrix through hydrogen bonding and hydrophobic interaction, effectively slowing down the oxidative degradation rate of luteolin in an acidic storage environment [44]. Similar studies have also reported that covalent binding between proteins and polyphenols (e.g., catechins, EGCG, or epigallocatechin gallate) can confer stronger DPPH free radical scavenging activity to the complexes than natural protein themselves [52,53,54].

As shown in Figure 3b, a significant concentration- and time-dependent enhancement of ABTS radical scavenging activity was observed in the casein–luteolin complexes. During the initial storage period (day 1), as the luteolin concentration increased from 0.02% to 0.06%, and the ABTS clearance rate rose significantly from 30.15% to 45.18%. Throughout the entire storage period, the ABTS scavenging capacity of all samples continued to improve, with the 0.06% luteolin group demonstrating the most prominent effect. By day 21, the ABTS clearance rate of the group treated with 0.06% luteolin increased by 18.82% compared to the first day. Among all test groups, the samples treated with 0.06% luteolin exhibited the highest and most stable antioxidant activity. This result indicates that the moderate addition of luteolin not only enhances the initial antioxidant performance of the complex but also leads to a significant improvement in its antioxidant stability over the storage period. During the initial storage period, some of the luteolin binds tightly to casein through non-covalent bonds, temporarily masking the phenolic hydroxyl group; as the refrigeration time prolongs, some binding sites slowly dissociate in acidic environments, releasing more free active phenolic hydroxyl groups. Previous studies reported that the complex prepared with hydroxytyrosol at 70 μmol/g exhibited an ABTS radical scavenging capacity of 58.69 ± 0.47%, which was 26.35% higher than that of the control (*p* < 0.05) [55].

### 3.6. In Vitro Protein Digestion Analysis of All Fermented Samples

**Figure 4:**
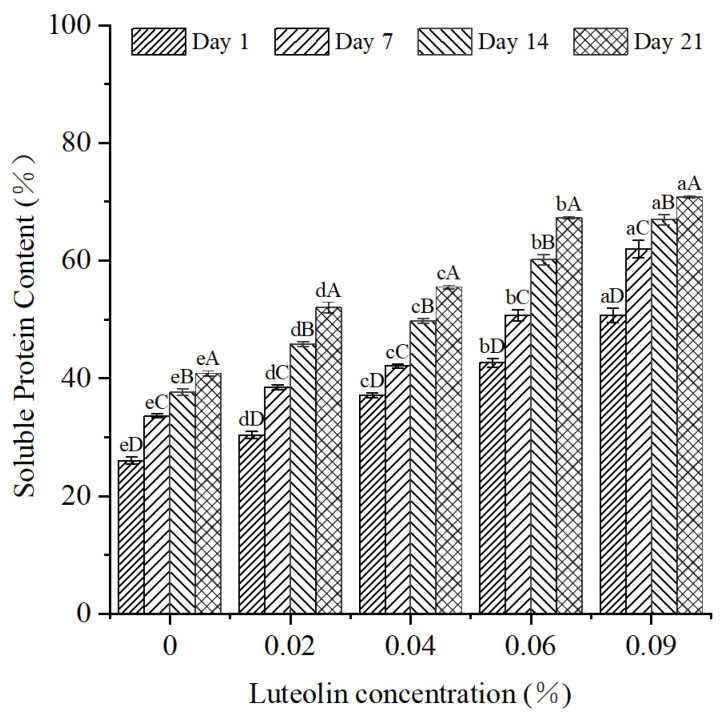
Evaluation of in vitro protein digestibility of all fermented samples. Note: Data were analyzed by two-way ANOVA. Values with different letters in the figure are significantly different (*p* < 0.05). The control sample is without luteolin.

Good protein digestibility is a key consideration in the development of new protein-rich foods, which aims to provide a healthier and more sustainable food supply chain. As illustrated in Figure 4, the interaction between luteolin and casein exerts a significant impact on the digestive properties of the protein. Specifically, with the luteolin concentration increased, the protein digestibility of the fermented sample rich luteolin increased significantly, and all luteolin-supplemented groups exhibited notably higher digestibility than the control group (*p* < 0.05). Throughout the 21-day refrigeration period, the in vitro digestibility of samples containing 0.06% luteolin showed a gradual upward trend, rising from 42.62% on day 1 to 67.28% on day 21. At the end of the storage period (day 21), the digestion rate of the fermented sample with 0.06% luteolin was significantly higher than that of the control fermented sample (40.86%), with an absolute difference of 26.42 percentage points (*p* < 0.05). These results indicate that luteolin not only does not inhibit casein digestion but also exerts a positive effect in promoting casein hydrolysis. The potential mechanism may be attributed to the non-covalent binding between luteolin and casein, which induces a moderate expansion of the protein’s secondary structure. This expansion facilitates the exposure of cleavage sites for pepsin and trypsin, thereby enhancing enzymatic hydrolysis efficiency [38]. Additionally, as a polyphenolic compound, luteolin may to a certain extent inhibit protein aggregation and precipitation in the digestive environment by forming soluble complexes with casein, thereby further improving overall protein digestibility [56].

### 3.7. Zeta Potential and Particle Size Analysis of All Samples

**Figure 5:**
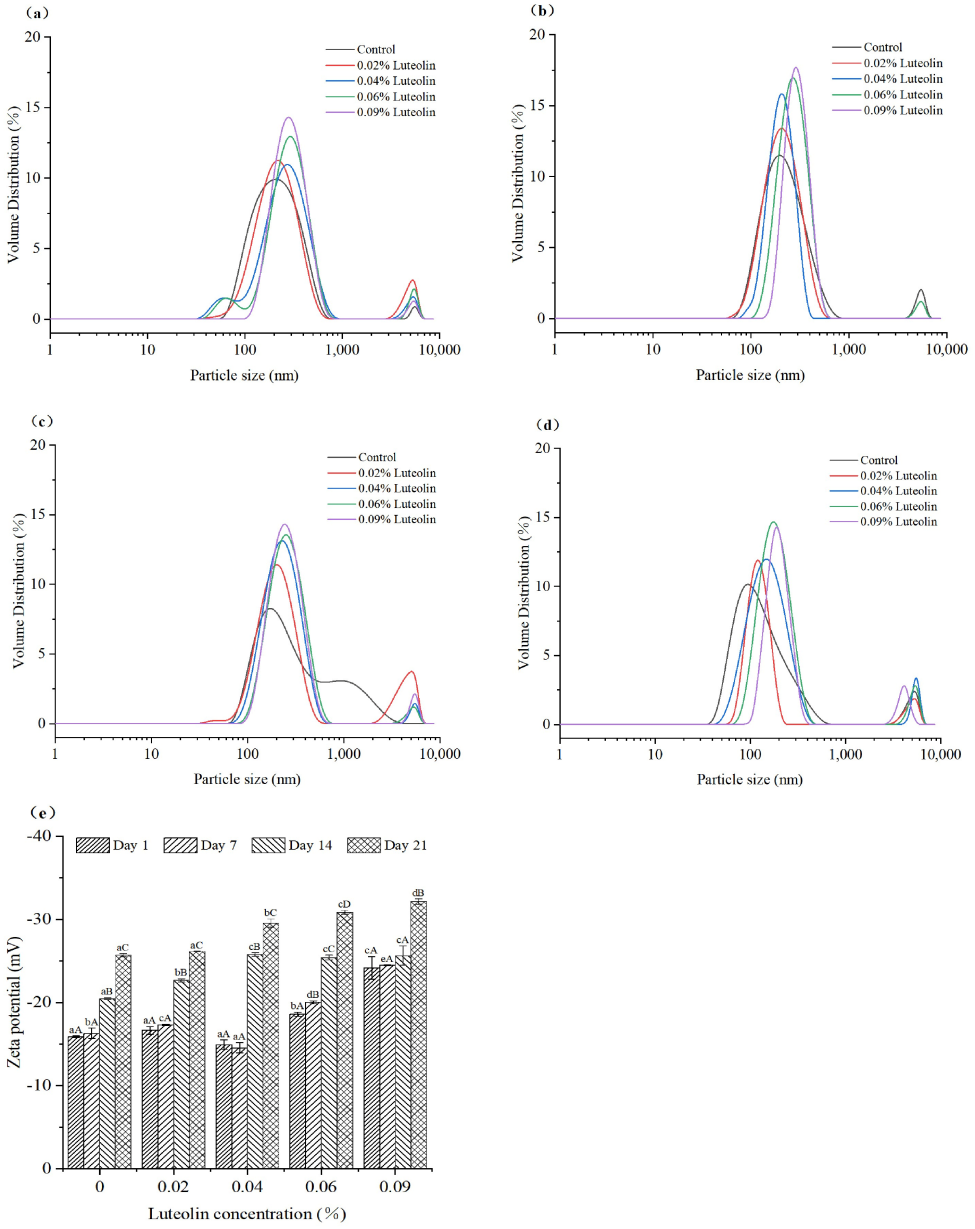
Size and Zeta potential changes in all samples. Particle size of all sample measured at 1 (**a**), 7 (**b**), 14 (**c**), and 21 days (**d**). (**e**) Zeta potential of all sample measured at 1 (**a**), 7 (**b**), 14 (**c**), and 21 days (**d**). Note: Data were analyzed by two-way ANOVA. Values with different letters in the figure are significantly different (*p* < 0.05). The control sample is without luteolin.

The particle size variation in the sample with added luteolin is illustrated in Figure 5. Results revealed that as luteolin level increased, the average particle size of the sample exhibited a significant upward trend (*p* < 0.05). On the first day of storage, the particle size of sample with 0.06% luteolin added elevated from 220 nm in the control group to approximately 295 nm. This phenomenon suggests that the multiple hydroxyl groups in the luteolin molecule can simultaneously form hydrogen bonds with different casein molecules, acting as “molecular bridges” to facilitate protein aggregation. Existing studies have clearly confirmed that phenolic compounds including tannic acid, epigallocatechin gallate (EGCG), and quercetin can promote casein aggregation and reduce its solubility [57]. Throughout the storage period, the particle size of all samples showed a gradual downward trend. The particle size of the sample containing 0.06% luteolin was approximately 295 nm on the first day and further decreased to around 164 nm on the 21st day. This observation implies that the sample may have undergone further structural rearrangement and densification during storage. As storage time extends, the interaction between polyphenols and proteins gradually reaches a state of equilibrium, thus allowing the formation of a more compact and ordered particle structure [58].

The changes in zeta potential of all samples are depicted in Figure 5e. Throughout the storage period, as the concentration of luteolin increased, the absolute value of the sample’s zeta potential showed a gradual upward trend. After storage for approximately 21 days, the zeta potential of the sample in the 0.06% luteolin group decreased to around −30.83 mV, in contrast to −25.68 mV in the control group. Compared with the zeta potential on day 1 (−18.58 mV), this represented a decrease of 12.25 mV. This variation indicates that the binding of luteolin enhances the negative charge density on the surface of casein molecules. This phenomenon can be attributed to modifications in molecular structure that alter the orientation and conformation of molecules at the solution interface, thereby enhancing the overall negative charge characteristics of the system [59]. This reflects the protein conformational rearrangement induced by luteolin: luteolin binding promotes the folding of hydrophobic regions within casein molecules, exposing negatively charged phosphoserine clusters to the outside, thereby significantly enhancing surface negative charge density while particle densification occurs. The higher electrostatic repulsion effectively inhibits excessive aggregation or precipitation during storage [60].

### 3.8. FT-IR Infrared Spectroscopy Analysis of All Samples

**Figure 6:**
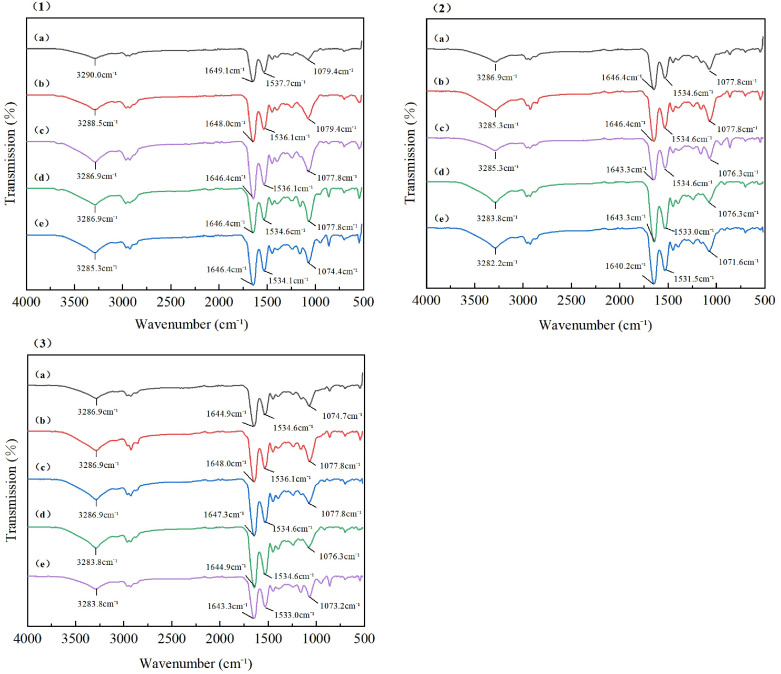
FTIR spectra results of all samples. FTIR spectra results of all samples measured at 1 (**1**), 14 (**2**), and 21 days (**3**). Control sample without luteolin (a), the sample containing 0.02% Luteolin (b), the sample containing 0.04% Luteolin (c), the sample containing 0.06% Luteolin (d),and the sample containing 0.09% Luteolin (e).

Variations in protein secondary conformations are typically reflected by two characteristic infrared absorption regions. The amide I band (1700–1600 cm^−1^) is assigned to the C=O stretching vibration of peptide bonds, while the amide II band (1600–1500 cm^−1^) originates from the coupling of N-H bending and C-N stretching vibrations [61]. Interactions between proteins and polyphenols alter the peak position and absorption intensity of these two characteristic bands, which precisely characterize conformational variations in protein secondary structures. The Fourier transform infrared spectroscopy (FTIR) spectra of all samples are presented in Figure 6. The spectral displacements of amide I and amide II bands confirmed that luteolin interacted with casein via C=O, N-H, and C-N functional groups, inducing secondary structural modifications of casein. The redshift of the amide I band toward lower wavenumbers was associated with reduced α-helix content and elevated proportions of β-sheets and random coils. This phenomenon was primarily attributed to the disruption of the hydrogen-bonding network surrounding peptide bonds, which was triggered by the binding interaction between casein and luteolin through C=O, C-N, and N-H functional groups. Furthermore, spectral variations observed at 3300 cm^−1^ further verified that hydrogen bonding served as the predominant intermolecular force mediating the interaction between casein and luteolin [62]. As a polyphenolic ligand, luteolin binds to casein via hydrogen bonding and hydrophobic interactions, further triggering conformational rearrangement of casein. Consistent with the present findings, Zhao et al. [61] reported that casein interacted with tannic acid and gallic acid through C=O, C-N, and N-H groups, whereas collagen mainly relied on C-N and N-H groups for polyphenol binding. However, when the storage period was extended to 21 days, the amide I band shifted toward higher wavenumbers. During long-term storage, homogeneous complexes underwent macroscopic aggregation, causing partial rupture of intermolecular hydrogen bonds and the expulsion of interfacial water molecules. These changes reduced the local polarity of C=O groups, thereby resulting in an upward wavenumber shift [63].

## 4. Conclusions

This study systematically explored the modulatory effects of luteolin on the structural characteristics, physicochemical properties, processing adaptability, antioxidant activity, and in vitro digestive performance of casein-based matrices. The results demonstrated that 0.06% luteolin supplementation effectively optimized the texture and viscoelasticity of casein composites and improved the overall quality and storage stability of dairy systems. Increasing luteolin dosage concentration dependently enhanced the radical scavenging ability of the matrix, which maintained stable antioxidant activity during 21 days of cold storage. Moreover, luteolin fortification optimized the gastrointestinal tolerance and digestive accessibility of casein aggregates, thereby improving the in vitro digestibility of the composite system. Luteolin supplementation altered the particle size and surface zeta potential of casein colloids. Specifically, luteolin spontaneously binds to casein through hydrogen bonding and hydrophobic interactions, inducing protein secondary structural rearrangement and microstructural reconstruction. Such molecular-level variations are the essential mechanism responsible for the improved macroscopic quality and functional performance of luteolin-fortified casein systems. Importantly, the developed luteolin–casein complexes possess prominent practical potential for functional food development and industrial application. As a natural bioactive additive, luteolin requires no complicated pretreatment, and the compounding and fermentation processes established in this study are fully compatible with conventional dairy production equipment, confirming excellent food processing feasibility. Economically, the moderate effective dosage avoids excessive raw material investment. Meanwhile, the enhanced storage stability of modified dairy products reduces quality deterioration and commodity loss during circulation, endowing the product with favorable cost competitiveness. Accordingly, luteolin–casein complexes can serve as efficient natural functional fortifiers and quality improvers, which have broad application prospects in novel functional fermented dairy products. In conclusion, luteolin can act as an effective natural structural and nutritional modifier to improve the overall quality and functional diversity of casein-based dairy matrices. This study clarifies the core structure–function relationship of luteolin–casein binary complexes, providing clear theoretical guidance for the development of polyphenol-fortified functional dairy foods. Nevertheless, this work has certain limitations. The structural analysis of protein complexes solely depends on FTIR spectroscopy, and the lack of multi-dimensional verification techniques, including Raman spectroscopy, X-ray diffraction, circular dichroism and transmission electron microscopy, makes the microstructural explanation relatively indirect. Future research will integrate multiple spectral characterization technologies and molecular dynamics simulations to further explore the precise binding mode, intermolecular affinity and dynamic conformational changes between luteolin and different casein subunits, so as to supplement and refine the interaction mechanism of polyphenol–protein conjugation. Overall, this work provides reliable theoretical basis and feasible technical support for the industrial development of high-stability, high-nutrition functional fermented dairy products.

## Figures and Tables

**Table 1:** Changes in pH and TA of all fermented samples during storage period.

Parameters	Storage Time (Days)	Control	0.02% Luteolin	0.04% Luteolin	0.06% Luteolin	0.09% Luteolin
pH	1	4.39 ± 0.04 ^aA^	4.30 ± 0.07 ^aA^	4.17 ± 0.02 ^bA^	4.12 ± 0.02 ^bA^	4.06 ± 0.03 ^bA^
	7	4.24 ± 0.01 ^aB^	4.18 ± 0.06 ^aAB^	4.14 ± 0.03 ^abA^	4.07 ± 0.01 ^bB^	4.04 ± 0.03 ^bB^
	14	4.17 ± 0.02 ^aC^	4.14 ± 0.01 ^aB^	4.08 ± 0.01 ^bB^	4.02 ± 0.01 ^cC^	4.01 ± 0.01 ^cC^
	21	4.08 ± 0.00 ^aD^	4.08 ± 0.02 ^aB^	4.04 ± 0.01 ^bB^	4.02 ± 0.05 ^bC^	3.98 ± 0.02 ^cD^
Titratable acidity (°T)	1	60.46 ± 7.33 ^bA^	65.93 ± 8.80 ^abA^	71.46 ± 0.41 ^abC^	75.60 ± 0.52 ^aC^	79.23 ± 0.55 ^aD^
	7	65.13 ± 3.44 ^cB^	70.13 ± 1.41 ^cA^	78.56 ± 1.71 ^bB^	82.13 ± 2.83 ^abB^	86.92 ± 2.59 ^aC^
	14	65.73 ± 1.02 ^dC^	74.06 ± 2.08 ^cAB^	87.05 ± 1.04 ^bA^	90.08 ± 1.37 ^abA^	91.88 ± 1.44 ^aB^
	21	66.66 ± 0.23 ^dD^	83.60 ± 2.61 ^cA^	89.62 ± 0.98 ^bA^	93.82 ± 2.10 ^abA^	95.87 ± 0.27 ^aA^

All values are expressed as mean ± SD (*n* = 3). ^a–d^ Means within the same row with different lowercase superscript letters denote a significant differences for the respective parameter (*p* < 0.05). Correspondingly, ^A–D^ means in the same column with distinct uppercase superscript letters indicating significant differences (*p* < 0.05).

**Table 2:** Color changes in these fermented samples during storage.

Parameters	Samples					
	Storage Time (Days)	Control Sample	Sample with 0.02% Luteolin	Sample with 0.04% Luteolin	Sample with 0.06% Luteolin	Sample with 0.09% Luteolin
L*	1	26.98 ± 0.04 ^bA^	24.55 ± 0.07 ^aB^	22.92 ± 0.02 ^aC^	18.88 ± 0.02 ^cD^	17.97 ± 0.03 ^cD^
	7	26.28 ± 0.01 ^bB^	23.00 ± 0.06 ^bC^	21.33 ± 0.03 ^aD^	16.80 ± 0.01 ^cE^	15.97 ± 0.03 ^cE^
	14	25.60 ± 0.02 ^cC^	22.27 ± 0.01 ^bD^	20.61 ± 0.01 ^aE^	15.26 ± 0.01 ^dF^	15.02 ± 0.01 ^dF^
	21	25.22 ± 0.00 ^cD^	20.49 ± 0.02 ^cE^	18.57 ± 0.01 ^bF^	14.61 ± 0.05 ^dG^	14.15 ± 0.02 ^eG^
a*	1	−0.14 ± 0.08 ^aA^	−0.35 ± 0.02 ^bB^	−0.53 ± 0.07 ^cC^	−0.54 ± 0.01 ^cC^	−0.67 ± 0.04 ^cD^
	7	−0.25 ± 0.03 ^aB^	−0.41 ± 0.05 ^bC^	−0.57 ± 0.03 ^cD^	−0.66 ± 0.01 ^cE^	−0.70 ± 0.07 ^cE^
	14	−0.27 ± 0.01 ^aB^	−0.45 ± 0.02 ^bD^	−0.58 ± 0.00 ^cD^	−0.71 ± 0.01 ^dE^	−0.77 ± 0.00 ^eE^
	21	−0.32 ± 0.01 ^aC^	−0.49 ± 0.01 ^bE^	−0.57 ± 0.01 ^cD^	−0.73 ± 0.00 ^dE^	−0.78 ± 0.01 ^eE^
b*	1	7.18 ± 0.81 ^bA^	8.40 ± 0.06 ^aB^	8.51 ± 0.25 ^aB^	8.71 ± 0.38 ^aB^	9.11 ± 0.86 ^aB^
	7	7.10 ± 0.18 ^cA^	8.41 ± 0.28 ^bB^	8.77 ± 0.16 ^aB^	9.25 ± 0.53 ^aB^	10.29 ± 0.68 ^aC^
	14	7.48 ± 0.03 ^eA^	8.72 ± 0.03 ^dB^	8.90 ± 0.05 ^cB^	9.64 ± 0.00 ^bC^	10.35 ± 0.02 ^aC^
	21	7.61 ± 0.07 ^eA^	9.07 ± 0.01 ^dC^	9.25 ± 0.01 ^cC^	10.23 ± 0.06 ^bD^	10.64 ± 0.01 ^aD^

All values are presented as mean ± SD (*n* = 3). ^a–e^ Means in the same row with different lowercase superscript letters demonstrate significant differences for the corresponding parameter (*p* < 0.05). Similarly, ^A–G^ means in the same column bearing different uppercase superscript letters indicate asignificant differences (*p* < 0.05). The control sample is without luteolin.

**Table 3:** Changes in syneresis of all fermented samples throughout the entire storage period.

Parameters	Storage Time (Days)	Control Sample	Sample with 0.02% Luteolin	Sample with 0.04% Luteolin	Sample with 0.06% Luteolin	Sample with 0.09% Luteolin
Syneresis (%)	1	34.61 ± 1.13 ^aB^	31.76 ± 0.65 ^bB^	28.64 ± 0.81 ^cC^	26.01 ± 0.59 ^dB^	24.52 ± 0.95 ^dB^
	7	36.29 ± 0.35 ^aAB^	34.07 ± 0.41 ^bAB^	31.16 ± 0.88 ^cB^	25.44 ± 0.69 ^dB^	24.47 ± 1.16 ^dB^
	14	37.67 ± 0.76 ^aAB^	36.05 ± 0.64 ^aA^	32.45 ± 0.60 ^bB^	30.01 ± 1.54 ^bA^	25.52 ± 1.23 ^cB^
	21	39.28 ± 2.04 ^aA^	37.22 ± 3.09 ^aA^	34.71 ± 0.56 ^abA^	32.44 ± 1.12 ^bA^	31.33 ± 0.32 ^bA^

All data are displayed as mean ± SD (*n* = 3). ^a–d^ Means in the same row with different superscript lowercase letters indicate a significant difference for that parameter (*p* < 0.05). Similarly, ^A–C^ means in the same column with different superscript uppercase letters indicate a significant difference (*p* < 0.05). The control sample is without luteolin.

**Table 4:** Texture changes in all fermented samples.

Parameters	Samples					
	Storage Time (Days)	Control Sample	Sample with 0.02% Luteolin	Sample with 0.04% Luteolin	Sample with 0.06% Luteolin	Sample with 0.09% Luteolin
Hardness (N)	1	0.27 ± 0.01 ^eC^	0.47 ± 0.18 ^dC^	0.71 ± 0.16 ^cB^	1.00 ± 0.03 ^aB^	0.87 ± 0.02 ^bC^
	7	0.32 ± 0.01 ^eB^	0.54 ± 0.02 ^dB^	0.81 ± 0.02 ^cA^	1.04 ± 0.00 ^aB^	0.92 ± 0.00 ^bB^
	14	0.34 ± 0.03 ^eAB^	0.58 ± 0.00 ^dA^	0.87 ± 0.01 ^cA^	1.17 ± 0.04 ^aA^	1.07 ± 0.00 ^bA^
	21	0.37 ± 0.01 ^eA^	0.54 ± 0.00 ^dB^	0.73 ± 0.01 ^cB^	1.18 ± 0.01 ^aA^	0.85 ± 0.03 ^bC^
Springiness (mm)	1	3.56 ± 0.00 ^eD^	4.54 ± 0.02 ^dD^	5.19 ± 0.01 ^cB^	6.97 ± 0.00 ^aC^	6.31 ± 0.01 ^bC^
	7	4.06 ± 0.07 ^dC^	5.45 ± 0.10 ^cC^	5.90 ± 0.61 ^bcB^	7.66 ± 0.02 ^aB^	6.33 ± 0.03 ^bC^
	14	4.25 ± 0.11 ^eB^	6.23 ± 0.01 ^dB^	7.21 ± 0.01 ^cA^	7.91 ± 0.06 ^aAB^	7.58 ± 0.04 ^bB^
	21	4.64 ± 0.00 ^eA^	6.53 ± 0.01 ^dA^	6.94 ± 0.02 ^cA^	8.33 ± 0.04 ^aA^	7.12 ± 1.28 ^bA^
Adhesiveness (N.mm)	1	0.23 ± 0.00 ^dD^	0.35 ± 0.00 ^cD^	0.46 ± 0.00 ^bC^	0.84 ± 0.03 ^aD^	0.81 ± 0.01 ^aC^
	7	0.28 ± 0.00 ^eC^	0.42 ± 0.00 ^dC^	0.61 ± 0.01 ^cB^	1.05 ± 0.01 ^aC^	0.72 ± 0.01 ^bB^
	14	0.34 ± 0.00 ^dB^	0.64 ± 0.01 ^cB^	1.02 ± 0.00 ^bA^	1.12 ± 0.03 ^aB^	1.06 ± 0.02 ^bA^
	21	0.48 ± 0.01 ^eA^	0.87 ± 0.00 ^dA^	1.02 ± 0.02 ^cA^	1.25 ± 0.01 ^aA^	1.06 ± 0.2 ^bA^

All experimental data are represented as mean ± SD (*n* = 3). ^a–e^ Means in the same row with different superscript lowercase letters demonstrate a significant difference for that parameter (*p* < 0.05). Similarly, ^A–D^ means in the same column with different superscript uppercase letters indicate a significant difference (*p* < 0.05). The control sample is without luteolin.

## Data Availability

The original contributions presented in this study are included in the article. Further inquiries can be directed to the corresponding authors.

## References

[1] Van de Langerijt T.M., O’Mahony J.A., Crowley S.V. (2023). Structural, binding and functional properties of milk protein-polyphenol systems: A review. Molecules.

[2] Sun X., Sarteshnizi R.A., Udenigwe C.C. (2022). Recent advances in protein–polyphenol interactions focusing on structural properties related to antioxidant activities. Curr. Opin. Food Sci..

[3] Tang Q., Roos Y.H., Miao S. (2024). Structure, gelation mechanism of plant proteins versus dairy proteins and evolving modification strategies. Trends Food Sci. Technol..

[4] Faccia M., Natrella G. (2024). Dairy Products: Processing Technology and Sensory Properties. Foods.

[5] Feng Y., Jin C., Lv S., Zhang H., Ren F., Wang J. (2023). Molecular Mechanisms and Applications of Polyphenol-Protein Complexes with Antioxidant Properties: A Review. Antioxidants.

[6] Mohanty D.P., Mohapatra S., Misra S., Sahu P.S. (2016). Milk derived bioactive peptides and their impact on human health—A review. Saudi J. Biol. Sci..

[7] Rudrapal M., Khairnar S.J., Khan J., Dukhyil A.B., Ansari M.A., Alomary M.N., Alshabrmi F.M., Palai S., Deb P.K., Devi R. (2022). Dietary Polyphenols and Their Role in Oxidative Stress-Induced Human Diseases: Insights Into Protective Effects, Antioxidant Potentials and Mechanism(s) of Action. Front. Pharmacol..

[8] Zhang H., Tsao R. (2016). Dietary polyphenols, oxidative stress and antioxidant and anti-inflammatory effects. Curr. Opin. Food Sci..

[9] Qu G., Chen J., Guo X. (2018). The beneficial and deleterious role of dietary polyphenols on chronic degenerative diseases by regulating gene expression. Biosci. Trends.

[10] Zeb A. (2020). Concept, mechanism, and applications of phenolic antioxidants in foods. J. Food Biochem..

[11] Sahraeian S., Rashidinejad A., Golmakani M.-T. (2024). Recent advances in the conjugation approaches for enhancing the bioavailability of polyphenols. Food Hydrocoll..

[12] Cao H., Saroglu O., Karadag A., Diaconeasa Z., Zoccatelli G., Conte-Junior C.A., Gonzalez-Aguilar G.A., Ou J., Bai W., Zamarioli C.M. (2021). Available technologies on improving the stability of polyphenols in food processing. Food Front..

[13] Oliveira G., Volino-Souza M., Conte-Júnior C.A., Alvares T.S. (2021). Food-derived polyphenol compounds and cardiovascular health: A nano-technological perspective. Food Biosci..

[14] Mao T., Wescombe P., Mohan M.S. (2023). Predominance of non-covalent interactions of polyphenols with milk proteins and their health promoting properties. Crit. Rev. Food Sci. Nutr..

[15] Ashwar B.A., Gani A. (2021). Noncovalent Interactions of Sea Buckthorn Polyphenols with Casein and Whey Proteins: Effect on the Stability, Antioxidant Potential, and Bioaccessibility of Polyphenols. ACS Food Sci. Technol..

[16] Bié J., Sepodes B., Fernandes P.C.B., Ribeiro M.H.L. (2023). Polyphenols in Health and Disease: Gut Microbiota, Bioaccessibility, and Bioavailability. Compounds.

[17] Ebrahimi P., Lante A., Grossmann L. (2025). Protein-polyphenol complexation vs. conjugation: A review on mechanisms, functional differences, and antioxidant-emulsifier roles. Food Hydrocoll..

[18] Ferraro V., Madureira A.R., Fonte P., Sarmento B., Gomes A.M., Pintado M.E. (2015). Evaluation of the interactions between rosmarinic acid and bovine milk casein. RSC Adv..

[19] Chen N., Xie K., Jiao Z., Zhang W., Deng H., Ashaolu T.J., Cheng K., Zhao C. (2025). Milk protein modulates antioxidant activity and metabolome stability in coffee beverages during thermal processing. J. Dairy Sci..

[20] Li Z., Kang S., Shu Q., Al-Wraikat M., Hao C., Liu Y. (2024). Structural modification and functional improvement of lactoferrin through non-covalent and covalent binding to coffee polyphenol. Innov. Food Sci. Emerg. Technol..

[21] Singh A., Yadav S., Pathak P., Verma A., Yadav J.P. (2024). Harnessing Luteolin’s therapeutic potential in human disorders: Medicinal significance, biological, clinical properties and analytical aspects. Pharmacol. Res.-Mod. Chin. Med..

[22] Caporali S., De Stefano A., Calabrese C., Giovannelli A., Pieri M., Savini I., Tesauro M., Bernardini S., Minieri M., Terrinoni A. (2022). Anti-Inflammatory and Active Biological Properties of the Plant-Derived Bioactive Compounds Luteolin and Luteolin 7-Glucoside. Nutrients.

[23] Yazar M., Sevindik M., Polat A.O., Koçer O., Kuşçu Karatepe H., Uysal İ. (2024). General properties, biosynthesis, pharmacological properties, biological activities and daily uses of luteolin. Prospect. Pharm. Sci..

[24] Abdrabou R., Salama R., El-Naga R., Azab S. (2024). The Protective Properties of Luteolin: A Comprehensive Review. Arch. Pharm. Sci. Ain Shams Univ..

[25] Alshehri S., Imam S.S., Altamimi M.A., Hussain A., Shakeel F., Elzayat E., Mohsin K., Ibrahim M., Alanazi F. (2020). Enhanced Dissolution of Luteolin by Solid Dispersion Prepared by Different Methods: Physicochemical Characterization and Antioxidant Activity. ACS Omega.

[26] Miyashita A., Ito J., Parida I.S., Syoji N., Fujii T., Takahashi H., Nakagawa K. (2022). Improving water dispersibility and bioavailability of luteolin using microemulsion system. Sci. Rep..

[27] Punia Bangar S., Kajla P., Chaudhary V., Sharma N., Ozogul F. (2023). Luteolin: A flavone with myriads of bioactivities and food applications. Food Biosci..

[28] Rauf A., Wilairatana P., Joshi P.B., Ahmad Z., Olatunde A., Hafeez N., Hemeg H.A., Mubarak M.S. (2024). Revisiting luteolin: An updated review on its anticancer potential. Heliyon.

[29] Lasik A., Pikul J., Majcher M., Lasik-Kurdyś M., Konieczny P. (2016). Characteristics of fermented ewe’s milk product with an increased ratio of natural whey proteins to caseins. Small Rumin. Res..

[30] Megrous S., Al-Dalali S., Yang Z. (2024). Physicochemical and functional properties of yoghurt supplemented with bioactive low-molecular-weight casein hydrolysates. Int. Dairy J..

[31] Parvarei M.M., Fazeli M.R., Mortazavian A.M., Nezhad S.S., Mortazavi S.A., Golabchifar A.A., Khorshidian N. (2021). Comparative effects of probiotic and paraprobiotic addition on microbiological, biochemical and physical properties of yogurt. Food Res. Int..

[32] Chen S., Hou G., Liu Q., Zhang Y., Liu Z., Mu H., Li Y. (2025). Application of microgels as fat replacers and polyphenol carriers in Cheddar cheese. Food Hydrocoll..

[33] Varnaitė L., Keršienė M., Šipailienė A., Kazernavičiūtė R., Venskutonis P.R., Leskauskaitė D. (2022). Fiber-rich cranberry pomace as food ingredient with functional activity for yogurt production. Foods.

[34] Cheng J., Tang D., Yang H., Wang X., Zhu M., Liu X. (2021). The dose-dependent effects of polyphenols and malondialdehyde on the emulsifying and gel properties of myofibrillar protein-mulberry polyphenol complex. Food Chem..

[35] Trigueros L., Wojdylo A., Sendra E. (2014). Antioxidant activity and interactions protein-polyphenol in pomergranate (*Punica granatum* L.) yoghurt. J. Agric. Food Chem..

[36] Ren C., Xiong W., Peng D., He Y., Zhou P., Li J., Li B. (2018). Effects of thermal sterilization on soy protein isolate/polyphenol complexes: Aspects of structure, in vitro digestibility and antioxidant activity. Food Res. Int..

[37] López-Miranda S., Hernández-Sánchez P., Serrano-Martínez A., Hellín P., Fenoll J., Núñez-Delicado E. (2011). Effect of ripening on protein content and enzymatic activity of Crimson Seedless table grape. Food Chem..

[38] Li S., Luo M., Gao Z., Zhang Y., Demircan B., Yan W., McClements D.J. (2025). Effects of polyphenols on the functional properties, digestibility, and iron bioavailability of potato protein-iron complexes. Food Chem..

[39] Li J., Zhang Y., Nardin C. (2025). Curcumin-modified gelatin nanocomplexes as novel food foaming Agents: Impact on foamability and interfacial properties. Food Hydrocoll..

[40] Zhang K., Huang J., Wang D., Wan X., Wang Y. (2024). Covalent polyphenols-proteins interactions in food processing: Formation mechanisms, quantification methods, bioactive effects, and applications. Front. Nutr..

[41] Poojary M.M., Hellwig M., Henle T., Lund M.N. (2023). Covalent bonding between polyphenols and proteins: Synthesis of caffeic acid-cysteine and chlorogenic acid-cysteine adducts and their quantification in dairy beverages. Food Chem..

[42] Kim W., Wang Y., Selomulya C. (2024). Emerging technologies to improve plant protein functionality with protein-polyphenol interactions. Trends Food Sci. Technol..

[43] Cheng X., Zhu J., Chen Z., Wu Z., Zhang F., Wu C., Fan G. (2023). Color stability and degradation kinetics of anthocyanins in mulberry stirred yoghurt fermented by different starter cultures. Food Sci. Biotechnol..

[44] Sahraei A., Sahraei R. (2024). Revealing binding mechanism of β-casein to chrysin, apigenin, and luteolin and locating its binding pockets by molecular docking and molecular dynamics. Biochem. Biophys. Res. Commun..

[45] Yang J., Zhao Y., Shan B., Duan Y., Zhou J., Cai M., Zhang H. (2023). Study on the interaction and functional properties of Dolichos lablab L. protein-tea polyphenols complexes. Int. J. Biol. Macromol..

[46] Liu K., Zha X.-Q., Li Q.-M., Pan L.-H., Luo J.-P. (2021). Hydrophobic interaction and hydrogen bonding driving the self-assembling of quinoa protein and flavonoids. Food Hydrocoll..

[47] Hanuka-Katz I., Okun Z., Parvari G., Shpigelman A. (2022). Structure dependent stability and antioxidant capacity of strawberry polyphenols in the presence of canola protein. Food Chem..

[48] Pei Y., Yuan L., Zhou W., Yang J. (2025). Tyrosinase-catalyzed soy protein and tannic acid interaction: Effects on structural and rheological properties of complexes. Gels.

[49] Gong W., Guo X.L., Wang S.J., Huang H.B., Zhu X.M. (2023). Construction of high internal phase Pickering emulsions using cold plasma modified soy protein isolate-proanthocyanidin complex. Food Res. Int..

[50] Yildirim-Elikoglu S., Erdem Y.K. (2018). Interactions between milk proteins and polyphenols: Binding mechanisms, related changes, and the future trends in the dairy industry. Food Rev. Int..

[51] Czubinski J., Dwiecki K. (2017). A review of methods used for investigation of protein–phenolic compound interactions. Int. J. Food Sci. Technol..

[52] Wang S., Li X., Zhu J., Liu H., Liu T., Yu G., Shao M. (2021). Covalent interaction between high hydrostatic pressure-pretreated rice bran protein hydrolysates and ferulic acid: Focus on antioxidant activities and emulsifying properties. J. Agric. Food Chem..

[53] Djuardi A.U.P., Yuliana N.D., Ogawa M., Akazawa T., Suhartono M.T. (2020). Emulsifying properties and antioxidant activity of soy protein isolate conjugated with tea polyphenol extracts. J. Food Sci. Technol..

[54] Jiang J., Zhang Z., Zhao J., Liu Y. (2018). The effect of non-covalent interaction of chlorogenic acid with whey protein and casein on physicochemical and radical-scavenging activity of in vitro protein digests. Food Chem..

[55] Gu M., Shi J., Zhang B., Wang X., Wang X. (2024). Covalent modification of soy protein isolates by hydroxytyrosol: Effects on structural and functional properties of adducts. LWT-Food Sci. Technol..

[56] Qie X., Wu Y., Chen Y., Liu C., Zeng M., Qin F., He Z. (2021). Competitive interactions among tea catechins, proteins, and digestive enzymes modulate in vitro protein digestibility, catechin bioaccessibility, and antioxidant activity of milk tea beverage model systems. Food Res. Int..

[57] Chen L., Chen N., He Q., Sun Q., Gao M.-R., Zeng W.-C. (2022). Effects of different phenolic compounds on the interfacial behaviour of casein and the action mechanism. Food Res. Int..

[58] Li R., Zhang Z., Chen J., Li H., Tang H. (2023). Investigating of zein-gum arabic-tea polyphenols ternary complex nanoparticles for luteolin encapsulation: Fabrication, characterization, and functional performance. Int. J. Biol. Macromol..

[59] Ke C., Liu B., Dudu O.E., Zhang S., Meng L., Wang Y., Wei W., Cheng J., Yan T. (2023). Modification of structural and functional characteristics of casein treated with quercetin via two interaction modes: Covalent and non-covalent interactions. Food Hydrocoll..

[60] Mohammadian M., Salami M., Assadpour E., Jafari S.M. (2024). Curcumin-protein complexes: Technological and biological functionalities. Trends Food Sci. Technol..

[61] Zhao Q., Yu X., Zhou C., Yagoub A.E.A., Ma H. (2020). Effects of collagen and casein with phenolic compounds interactions on protein in vitro digestion and antioxidation. LWT-Food Sci. Technol..

[62] Meng Y., Li C. (2021). Conformational changes and functional properties of whey protein isolate-polyphenol complexes formed by non-covalent interaction. Food Chem..

[63] Dai S., Lian Z., Qi W., Chen Y., Tong X., Tian T., Jiang L. (2022). Non-covalent interaction of soy protein isolate and catechin: Mechanism and effects on protein conformation. Food Chem..

